# Tracking Global Fund HIV/AIDS resources used for sexual and reproductive health service integration: case study from Ethiopia

**DOI:** 10.1186/s12992-015-0106-z

**Published:** 2015-05-27

**Authors:** Sangeeta Mookherji, Samantha Ski, Dale Huntington

**Affiliations:** Department of Global Health, George Washington University Milken Institute of Public Health, 950 New Hampshire Ave, 4th Floor, 20052 Washington, DC USA; Asia Pacific Observatory on Health Systems and Policies, World Health Organization, Western Pacific Regional Office, Manila, Philippines

**Keywords:** Resource tracking, Global Fund, HIV, SRH, Diagonal financing, Service integration, Health systems strengthening, Ethiopia

## Abstract

**Objective/Background:**

The Global Fund to Fight AIDS, Tuberculosis & Malaria (GF) strives for high value for money, encouraging countries to integrate synergistic services and systems strengthening to maximize investments. The GF needs to show how, and how much, its grants support more than just HIV/AIDS, TB and malaria.

Sexual and Reproductive Health (SRH) has been part of HIV/AIDS grants since 2007. Previous studies showed the GF PBF system does not allow resource tracking for SRH integration within HIV/AIDS grants. We present findings from a resource tracking case study using primary data collected at country level.

**Methods:**

Ethiopia was the study site. We reviewed data from four HIV/AIDS grants from January 2009-June 2011 and categorized SDAs and activities as directly, indirectly, or not related to SRH integration. Data included: GF PBF data; financial, performance, in-depth interview and facility observation data from Ethiopia.

**Results:**

All HIV/AIDS grants in Ethiopia support SRH integration activities (12-100%). Using activities within SDAs, expenditures directly supporting SRH integration increased from 25% to 66% for the largest HIV/AIDS grant, and from 21% to 34% for the smaller PMTCT-focused grant. Using SDAs to categorize expenditures underestimated direct investments in SRH integration; activity-based categorization is more accurate.

The important finding is that primary data collection could not resolve the limitations in using GF GPR data for resource tracking. The remedy is to require existing activity-based budgets and expenditure reports as part of PBF reporting requirements, and make them available in the grant portfolio database. The GF should do this quickly, as it is a serious shortfall in the GF guiding principle of transparency.

**Conclusions:**

Showing high value for money is important for maximizing impact and replenishments. The Global Fund should routinely track HIV/AIDs grant expenditures to disease control, service integration, and overall health systems strengthening. The current PBF system will not allow this. Real-time expenditure analysis could be achieved by integrating existing activity-based financial data into the routine PBF system. The GF’s New Funding Model and the 2012-2016 strategy present good opportunities for over-hauling the PBF system to improve transparency and allow the GF to monitor and maximize value for money.

**Electronic supplementary material:**

The online version of this article (doi:10.1186/s12992-015-0106-z) contains supplementary material, which is available to authorized users.

## Background

The Global Fund to Fight AIDS, Tuberculosis & Malaria (GF) is a leading financier of global health initiatives. In 2012, the GF celebrated its 10^th^ anniversary, and by 2014, had committed more than USD 32 billion to grants in more than 150 countries. The majority of these funds support the three disease areas that the GF was launched to address: HIV/AIDS control activities (52%), followed by malaria (29%), and tuberculosis (15%) [1]. Traditionally, the GF has encouraged countries to integrate related, synergistic services in order to maximize investments, and has opened new grant channels to specifically support these areas. The GF grant portfolio currently funds both TB-HIV integration (2%) and health systems strengthening (HSS) (2%) [1].

The GF provides its financing through a performance-based funding (PBF) model – it tracks achievement of targets promised in the proposals, and disburses committed funds accordingly, with rewards for good performance, and some penalties for poor performance in the form of delays in receiving resources. The PBF model, which requires grant recipients to submit Grant Performance Reports (GPRs) on a regular basis, introduced a higher degree of transparency on the value of GF funding than previously, as the GF also committed to make data on grant progress publicly available. The large volume of GF funding, along with the PBF model and a grant-making approach that is country-led, have contributed to significant declines in deaths and infections from the three targeted diseases, and have helped forge a stronger link between funding and grant performance [2].

Pressing challenges to the current GF model are evident, despite successes. After an initial surge in funding to combat HIV/AIDS, tuberculosis, and malaria, the post-2008 economic climate has meant that financing streams have stabilized or diminished; this in turn has led to increasing demands that recipient countries allocate funds to demonstrably effective interventions, and provide co-financing. The GF recognized the necessity of revamping its investment strategy in 2011, articulated in its 2012-2016 strategy, “Investing for Impact”, where the concept of value for money is highly visible. Promoting and demonstrating maximized value for money presents a particular challenge for the GF, with its country-owned and led resource allocation model, and the integration and HSS it has encouraged within grants [2-5]. Showing high value for money ultimately will require the GF to estimate the value of investments made within its vertical, disease-targeted funds in synergistic areas, such service integration and health systems strengthening.

One such area is investment in Sexual and Reproductive Health (SRH). The GF has encouraged integration of within its HIV/AIDS grants since 2007, after SRH-HIV integration was put forth as a priority and best practice by the World Health Organization, World Bank, and others [6,7]. Efficiencies that can be gained through HIV-SRH program linkages and service integration offer one way to purchase more health for every dollar of GF investment, and multiple organizations have since produced guidance and technical support to countries on how to do this [6,8].

In 2009 and 2010, WHO conducted two studies to assess the progress on SRH-HIV integration in GF HIV/AIDS grants. Both used the existing GF grant portfolio database. The first examined the extent to which SRH programmatic elements had been included in GF HIV proposals and grant agreements [9]. The authors examined 134 approved HIV/AIDS proposals between Rounds 1 through 7 and found that four broad elements pertaining to SRH were included in 70% of the proposals, but were found in a lower proportion of the corresponding signed grant agreements. For example, the diagnosis and treatment of sexually transmitted infections (STIs) was found in 69% of the HIV-related proposals but in only 54% of the corresponding grant agreements. The second study assessed the extent to which SRH interventions can be monitored in Global Fund performance frameworks [10]. The authors examined grant performance report (GPR) data on 252 signed HIV/AIDS grants. They found that 94% of the HIV grant programs supported what looked to be SRH activities, but could gather little evidence on success in actual service integration or outcomes related to HIV-SRH integration, primarily due to inadequacy of SRH-related performance indicators included in the monitoring framework.

The combined results from these two studies showed that the data publicly available through GF Secretariat – including approved proposals, signed grant agreements, performance monitoring frameworks, GPRs, and expenditures by service delivery area (SDA) - even when examined together, do not allow tracking of resources allocated to SRH integration within HIV/AIDS grants. The increasing frustration with the limited understanding of SRH-HIV integration this data allowed prompted the WHO and GF to collaborate on a study that would track SRH integration resources by collecting data at country level. The hope was that a case study of one country would support the development of a methodology for tracking SRH-HIV resources that could be applied in other countries. Here we present the findings from the resulting country-level resource tracking study to understand investments in SRH integration made through GF HIV/AIDS grants in Ethiopia.

### Study purpose

This study intended to investigate the following questions:

1. In the selected proposals identified as including sexual and reproductive elements, what amount of funds was allocated to sexual and reproductive health activities in the corresponding grant agreement?
2. What has been the correspondence between the budget and selected expenditure categories for the SRH-related activities in the grant agreements?
3. Is there evidence that the additional funding has actually been allocated to sexual and reproductive health activities of the program that Global Fund grant(s) support?

## Methods

A case study methodology was developed to understand how investments supported by the Global Fund’s HIV/AIDS grants are used for sexual and reproductive health services at the country level. It was designed to build on the data available through Global Fund grant performance monitoring system while incorporating more detailed and varied data from country level. This study did not require ethical approval, as it used existing data. Informed consent for interviews was obtained verbally, using a letter of introduction from the GF (Additional file 1).

### Case selection

Ethiopia was selected as the study site from a short list of countries that receive significant amounts through GF HIV/AIDS grants and had strong references to SRH integration, as identified through the previous WHO studies in 2009 and 2010. As of 2014, the GF had committed 1.75 billion USD to Ethiopia, of which more than 1 billion USD (62%) is for HIV/AIDS grants. Three HIV/AIDS grants were made to Ethiopia in Round 7 under the proposal titled, “Ensuring Quality HIV/AIDS Services by Consolidating and Strengthening Existing HIV/AIDS Prevention, Treatment, Care and Support Programs.” Of these, only one was thought to include SRH activities after review of the grant proposal and agreements (ETH-708-G08-H; see Table 1), but all three Round 7 grants were included in this study. Consultation with GF clarified that the Rolling Continuation Channel (RCC) for the Round 2 grant (ETH-202-G03-H-00) should also be included. In all, four HIV/AIDS grants were included in this case study (Table 1).

**Table 1:** **Active Global Fund HIV/AIDS grants in Ethiopia, December 2011**

**Grant and PR**	**Grant number**	**Time period analyzed**	Amount requested*	Amount approved**	Amount disbursed***	Amount expended***
RCC HAPCO	ETH-202-G03-H-00	Jan 2009 – Jun 2011 (2.5 years) + (Life of Grant: Jan09 – Dec14; 6 years)	$273,882,281++ ($707,702,367)	$297,493,992 (109% of requested) ($435,001,702)	$179,234,054 (60% of approved)	$30,142,540 (17% of disbursed)
Round 7 HAPCO	ETH-708-G08-H	Jan 2009 – Dec 2010 (2 years) (Life of Grant: 3 years requested, 2 years approved – Jan09-Dec10)	$41,777,416 ($49,506,807)	$41,666,516 (100% of requested) ($41,666,516)	$35,728,140 (86% of approved)	$11,662,571 (33% of disbursed)
Round 7 EIFDDA	ETH-708-G09-H	Jan 2009 – Jun 2011 (2.5 years) (Life of Grant: Jan09-Dec13; 5 years)	$17,326,041++ ($31,997,188)	$17,546,192 (101% of requested) $30,177,691	$17,429,768 (99% of approved)	$13,774,229 (79% of disbursed)
Round 7 NEP+	ETH-708-G07-H	Apr 1, 2009 – Sep 2011 (2.5 years) (Life of Grant: Apr09-Mar13; 5 years)	$12,152,136++ ($24,757,588)	$12,177,176 (100% of requested) ($23,226,898+++)	$12,647,639 (104% of approved)	$11,068,009 (88% of disbursed)

^*^From grant proposal; ^**^From grant agreement, ^***^ Provided by the LFA.

^+^ Note: First disbursement not until September 2010.

^++^ Figure estimated from proposal budget using year one plus year two plus one half of year three amounts, as quarterly breakdown is not provided in proposal budget.

^+++^ Amount in grant agreement differs from amount provided in GF online grant portfolio financial overview.

These four grants approved a total of $331,358,836 for Ethiopia, the largest being the RCC grant to the government’s HIV/AIDS Prevention and Control Office (HAPCO). The Round 7 proposal titled, “Ensuring Quality HIV/AIDS Services by Consolidating and Strengthening Existing HIV/AIDS Prevention, Treatment, Care and Support Programs”, awarded smaller grants to three PRs: HAPCO, and two non-government organizations, the Ethiopian Interfaith Forum for Development Dialogue and Action (EIFDDA) and the Network of Networks of HIV Positives in Ethiopia (NEP+). We reviewed data from January 2009, when the grants began, through June 2011, as June 2011 marked the end of the most recent quarter for which expenditure data was available.

### Framework for categorizing costs and expenditures

**Figure 1:**
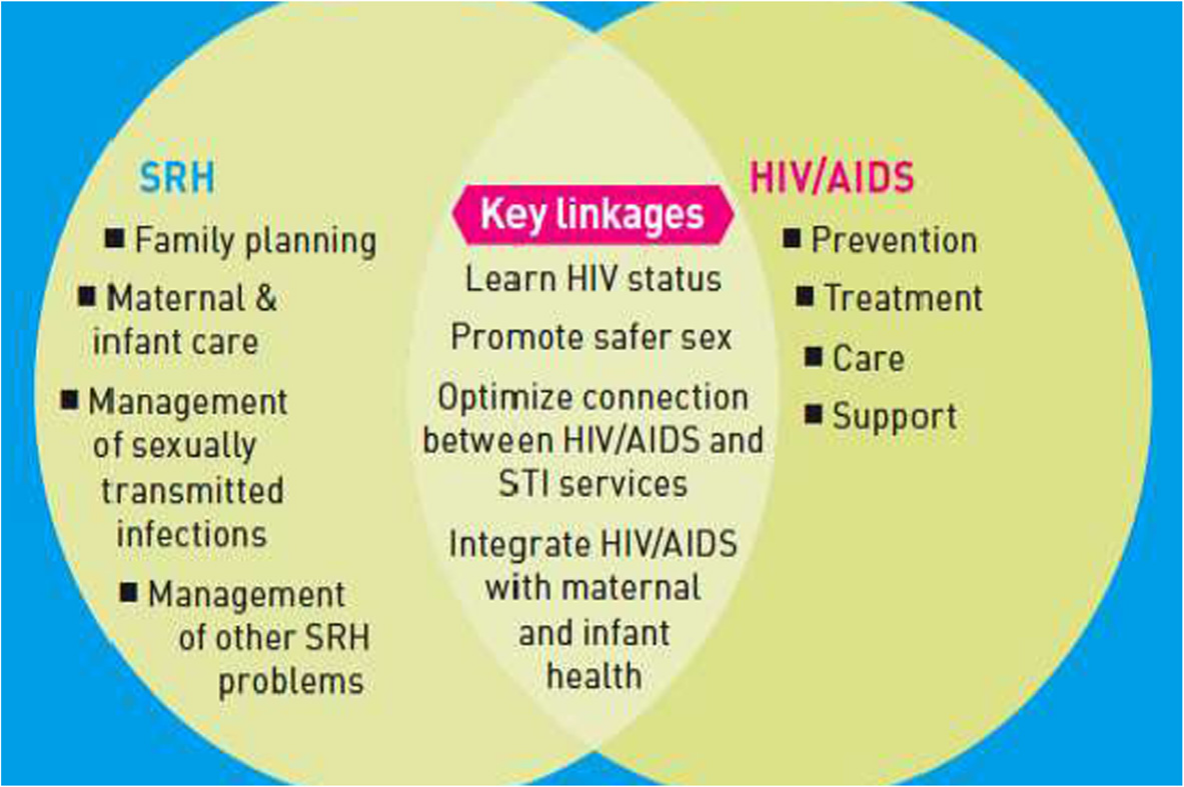
SRH-HIV integration framework.

As the purpose of the expenditure analysis was to assess investments in SRH made through Ethiopia’s active HIV/AIDS grants, we needed to allocate budget and expenditure amounts to SRH service linkage areas. We used the 2005 “A Framework for Priority Linkages” (Figure 1), in keeping with the approach used in earlier studies [9,10], to categorize HIV/AIDS grant activities as one of the following:

1. *Directly integration related*: program activities for SRH (family planning, maternal, neonatal and child health, sexually transmitted infections), HIV/AIDS (prevention only), as well as the key linkage areas in the 2005 Framework (HIV status awareness; promotion of safe sex; STI and HIV service connection; integration of HIV/AIDS into MNCH).
2. *Indirectly integration related*: activities contributing to health systems strengthening, such as human resources, supply chain, infrastructure and institution strengthening, and M&E
3. *Non-integration related*: activities supporting income-generating activities for PLWHA (IGA), blood safety, and opportunistic infections.

### Background information on GF financial data and reporting systems

Budget, disbursement, and expenditure data for GF grants are tracked by the Principal Recipients (PRs), who report to the Local Funding Agent (LFA), who reports to the GF Secretariat. Each of these three forms of financial data (budget, disbursement, and expenditure) contains costs at the level of Service Delivery Area (SDA), which are broad categories that contain many distinct activities (e.g., Behavior Change Communication or PMTCT), and may vary from year to year. For example, SDA 1.1, PMTCT, in ET-708-G08-H, includes 12 activities in Year 1, and nine in Year 2. SDA 9, Institutional Strengthening, in ET-202-G03-H-00, has five activities in year 1, four in Year 2, and two in Year 3 (tables a-d, Additional file 2).

**Figure 2:**
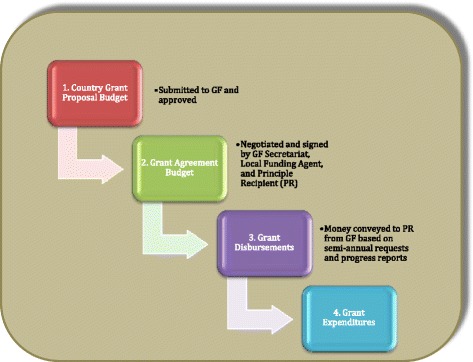
Financial data used to assess SRH integration in GF HIV/AIDS grants.

Detailed grant budgets are generated by PRs as a part of the proposal process and submitted for review as a part of the proposal. These budgets are then typically modified during the grant agreement negotiations, and an adjusted budget, by SDA, is included with the signed grant agreement (Figure 2). However, the signed budget by activity level is not included in the agreement, nor is it publicly available. These are kept by the Secretariat, PR, and LFA, and were made available to the study team not in original format, but as part of the first phase Budget Extraction by the GF Secretariat, and as expenditure reports by the LFA. For two grants in Ethiopia (ETH-202-G03-00 and ETH-708-G08-H), we were able to track expenditures by activities within SDAs.

The GF also requires PRs to report expenditures according to 8 pre-defined cost categories that it applies across it grant portfolio (Table 2). The Secretariat’s budget extraction included an analysis by these standard cost categories:

**Table 2:** **SRH Resource tracking in GF HIV grants, Ethiopia case study data collection**

**Data collection phase**	**Purpose**	**Dates**	**Data collected**	**Data sources**
**1**	Identify costs and performance indicators related to sexual and reproductive health in the selected country grants	June-September 2011	Budget allocations by SDA, activity and Cost Category	GF Secretariat; GF website
**2**	Obtain complete grant budget, disbursement, and expenditure data from LFA and PRs; understand program and budget allocation changes made between proposal and negotiated grant agreement; further specify through discussion with key informants which grant program activities can be considered SRH-integration related	September- November 2011	Grant budgets, disbursements and expenditures, by PR; by activity within SDAs for RCC; SRH and HIV/AIDS performance statistics; drugs and supplies data from PFSA; MNCH, RH, PMTCT, ANC, and HIV/AIDS policies and recent review reports; and National Health Accounts (NHA) data, including the HIV/AIDS and RH sub-accounts	LFA; grant PRs; FMOH; FHAPCO; PFSA Central; WHO; UNAIDS; UNFPA; UNICEF; other in-country partners
**3**	Further specify through observation of health facilities how SRH integration is realized, to understand impact of GF integration-related funds	December 2011	Regional HAPCO budgets, disbursements, and expenditures; regional SRH and HIV/AIDS performance data; Regional PFSA data; health facility integration maps	Amhara and Addis Ababa regions: 3 FMOH health centers in each; RHB; Regional PFSA Hubs

1. Communication materials
2. Health Products and Health Equipment
3. Human Resources
4. Infrastructure
5. Living Support to Clients
6. Medicines and Pharmaceutical Products
7. Monitoring and Evaluation
8. Overheads

GPRs report progress according to goals, objectives, and targets, by each performance indicator included in the grant agreement. Most of these targets, especially those set for the first 3 years of grant implementation, are outputs, and others reflect the results of several different activities, making the currently reported indicators difficult to use for assessing integration of SRH and HIV/AIDS. An indicator such as the numbers of condoms distributed is both a family planning and STI and HIV prevention activity, and therefore straightforward to interpret in terms of integration; numbers of people tested for HIV is more difficult to understand in terms of SRH integration unless the context of the target groups and test promotion strategies being used by the activities is considered. Indicators specific to SRH are not currently a part of the GF PBF.

### Data collection

Data collection proceeded in three phases (Table 2), of which only the first two were part of the original study plan. The third phase was added after it was confirmed that the data needed to improve expenditure categorization were not available at the central level in Ethiopia.

In the first phase, the GF Secretariat provided an initial budget extraction, by SDA, activity and Cost Category, using the final costed workplans that accompany the signing of the grant agreements, but which are not publicly available (the methodology used by the Secretariat is provided in Appendix A).

The second phase focused on data collection at the national level, in Ethiopia, including; budgets, disbursements and current expenditures, by PR and by activity within SDAs when possible. To provide context for the prioritized SRH activities identified in the expenditure reports, we also reviewed current and historical SRH and HIV/AIDS performance statistics; MNCH, RH, PMTCT, ANC, and HIV/AIDS policies and recent review reports; and National Health Accounts (NHA) data, including the HIV/AIDS and Reproductive Health sub-accounts. In addition, 28 in-depth interviews with key stakeholders were conducted primarily in the second phase of data collection to gain deeper understanding of the grant negotiation process, grant implementation challenges, SRH integration and PMTCT priorities, and how SRH, PMTCT and integration policies were being implemented through programs in general.

Financial data used for the resource tracking analysis were collected from three sources (GF Secretariat, Ethiopia’s Local Funding Agent (LFA), Ethiopia’s HIV/AIDS grant Principal Recipients) and checked for consistency across sources and reconciled when needed, before analysis. Reconciliation was rarely necessary because Ethiopia’s GF HIV/AIDS grants had recently been audited. Data from the LFA were used for analysis as it was in a consistent format across all three PRs. These data included: most current expenditures and disbursement reports, and negotiated budgets; grant performance reports, grant proposals, and grant agreements. Performance data for SRH and HIV/AIDS integration focused primarily on available PMTCT-related indicators, but also included condoms distributed, test kits distributed, and facilities provided with STI testing and treatment supplies. Most recent GPRs for each grant and annual FMOH reports generated (EFY 2001, 2002, and 2003) were used to identify GF HIV/AIDS grant contributions to overall SRH performance.

After reviewing available financial and program performance data, the study team recognized that a deeper understanding of the SRH, HIV/AIDS and GF grant negotiation processes was necessary, to boost confidence in how we categorized expenditures and estimates SRH integration investments. In particular, we needed further clarification on how expenditures were allocated within SDAs that included both SRH and non-SRH integration activities. Central-level key informants confirmed that the necessary information would likely be available at the health facility level, and encouraged the team to conduct facility visits to triangulate information on SRH-HIV integration.

Health facility visits in the third phase of data collection focused on two regions, Amhara and Addis Ababa. These were selected as positive deviant cases; Amhara because of demonstrated progress in integration (also because of the presence of development partners that are providing technical support to SRH-HIV integration), and Addis Ababa because of the substantial HIV burden. Three health facilities were selected from each region: 1 hospital and 2 health centers. In addition, program performance and expenditures data were collected from Regional Health Bureaus (RHBs), Regional HAPCOs, and Regional PFSAs, and interviews conducted with 21 additional key informants at regional and facility levels. The study team mapped the integration of SRH and HIV services at the facility level, as well as patient flow among the services. In addition, the study team conducted interviews with health facility managers, SRH and HIV service providers, and on some occasions, clients, to better understand how SRH-HIV integration was working and potentially offering increased efficiency of services and ease of navigation to beneficiaries and providers alike. This information was used in this study to refine and triangulate the categorization of SRH-related expenditures, in order to improve the estimation of GF investment being made through HIV/AIDS grants.

### Study findings

#### Budget and expenditure comparative analysis

A comparison of the proposal budgets, signed budgets, and expenditures by SRH-related category was conducted at the grant level to determine for which service delivery areas the budget was adjusted and how, as a part of the negotiation process (Tables a-d, Additional file 2). We could not identify appreciable differences between proposal amounts and budgeted amounts by SDA. We were unable to assess any budget adjustments to activities within SDAs, as there was not correspondence between costed SDAs and activities in the signed budgets, and the original proposals. We noted that none of the directly-related SRH integration categories seemed to be missing in the signed budgets, as compared to the proposals. We also noted that, of the four linkage areas, none of the four HIV/AIDS grants in Ethiopia seemed to address the third linkage area, “optimize connections between HIV/AIDS and STI services” (Table 3).

**Table 3:** Direct integration-related expenditures by key linkage area*, using activity categories

	**ET-202-G03-H-00**	**ET-708-G08-H**	**Total expenditures (% of directly integration-related expenditures for both grants)**
A. Learn HIV Status and Access Services	$4,089,481	$0	$4,089,481 (14.7%)
B. Promote Safer and Healthier Sex	$1,247,661	$0	$1,247,661 (4.5%)
C. Optimize the Connection Between HIV and STI Services	$0	$0	$0 (0%)
D. Integrate HIV/AIDS with Maternal and Infant Health	$0	$8,009,064	$8,009,064 (28.8%)
Multiple Linkage Areas+	$14,439,628	$0	$14,439,628 (52.0%)
TOTAL	$19,776,770	$8,009,064	$27,785,834

*Linkage areas based on the WHO et al. Sexual and Reproductive Health & HIV/AIDS: A Framework for Priority Linkages (2005);

+Two activities in the RCC grant involved multiple linkage areas.

When we tried to track expenditures according to the GF cost categories, we found many costs were categorized differently in PR records compared with budgets in proposals and costed activity-based workplan budgets. For example, Activity 2.1.3 (“Accommodation services for key health professionals and night duty staffs in 50 remote health centers”, in SDA 2), is categorized as Human Resources in the signed budget, but tracked as Infrastructure by the PR and LFA. This one activity accounted for 14% of the total budget amount for ET-708-G08-H, and so significantly affected comparisons by cost category that any interpretation of results was difficult.

#### Expenditure analysis for SRH investment

**Figure 3:**
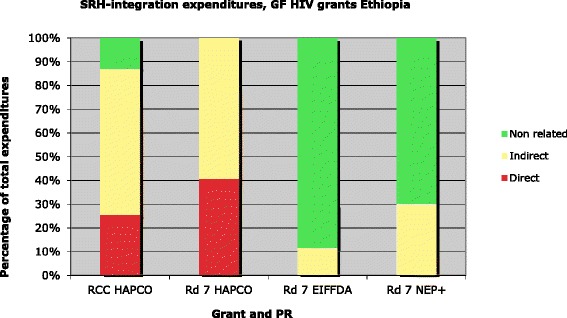
HIV grant expenditure distribution for SRH integration, by SDA, as of June 2011.

A large proportion of active Global Fund HIV-AIDS grants in Ethiopia are being expended on activities that support the integration of SRH services, either directly or indirectly (12-100%, Figure 3).

### Analysis by SDA

Using SDAs as the unit of analysis, all four active HIV/AIDS grants included activities that at least indirectly supported integration of SRH activities (12-62%, Figure 3), but only ETH-202-G03-H-00 and ET-708-G08-H included activities directly related to SRH integration (25% and 41%). The other two grants included only indirectly-related SRH integration activities; 12% and 30% of expenditures supported these SRH. Using SDAs, 100% of ET-708-G08-H grant expenditures and 88% of ETH-202-G03-H-00 grant expenditures were supporting SRH integration either directly or indirectly (Figure 3).

### Analysis by activity

We analyzed expenditure by activities within SDA, for two grants for which the data were available, and which were the only grants to include activities directly related to SRH integration: ETH-202-G03-H-00 and ET-708-G08-H. For ETH-202-G03-H-00, the proportion of expenditures directly supporting SRH integration increases from 25% to 66% when activities within SDAs are categorized according to SRH integration investment. For ET-708-G08-H, which is focused on PMTCT, the proportion of expenditures directly supporting SRH integration increase from from 21% to 34% when activities are the unit of analysis (Table 4).

**Table 4:** **Comparison of SRH integration investment, by SDA and by activity**

**Expenditures through June 2011:**	**Directly integration related**	**Indirectly integration related**	**Non integration related**	**Total**
ET-202-G03-H-00 (Source: LFA data)				
By SDA	$7,699,594	$18,562,575	$3,880,371	$30,142,540
	26%	62%	13%	
By Activity	$19,776,770	$6,485,399	$3,880,371	$30,142,540
	66%	22%	13%	
ET-708-G08-H (Source: PR data)				
By SDA	$6,889,224	$16,878,968	$0	$23,768,192
	21%	79%	0%	
By Activity	$8,009,062	$15,759,130	$0	$23,768,192
	34%	66%	0%	

Most of the re-categorization of expenditures stemmed from health systems strengthening activities hidden in SDAs that were assumed to be indirect investment in SRH integration, but upon examination of specific activities, turned out to include direct investment. For example, the major difference in ETH-202-G08-H-00 estimates of direct investment comes from an activity within SDA 9: Institutional Strengthening, originally categorized as indirect investment. But after it was found to include the activity, “Upgrading and equipping 187 rural health facilities to provide HCT, PMTCT and ART services,”, the expenditures for this activity within the SDA were re-categorized as direct investment, which more than doubled the grant total of direct SRH integration-related expenditures. The principal difference for ET-708-G08-H stems from the SDA “Human Resources”; breaking these expenditures down by activity showed that approximately 12% of the total expenditures within this SDA were direct, not indirect, investments in SRH-HIV integration.

## Discussion

We present our discussion under two themes. First, we discuss the interpretation of the resource tracking results, and then, perhaps more importantly, we discuss the experiences of conducting this resource tracking study, and implications for the GF and its ability to demonstrate and maximize value for money.

### Interpreting the study results

This study found that in Ethiopia, Global Fund HIV-AIDS grants provide substantial investment for both SRH integration. Changing the unit of analysis from SDA to activities significantly changed our estimations of direct v. indirect investment being made by Ethiopia’s GF HIV/AIDS grants in SRH integration. In the case of ETH-202-G08-H-00 and ET-708-G08-H, using SDAs underestimates the direct investment that GF HIV/AIDS grants make in SRH integration. There is no sound way to hypothesize whether this would be the case with other HIV/AIDS grants in Ethiopia, or in other countries; using SDAs could just as easily over-estimate investment in SRH-HIV integration, depending on how SDAs were written in the original grant proposal and affected in the grant negotiation process. In the absence of activity-specific expenditure availability, SDA-based expenditure analysis findings should be interpreted with great caution, in particular if there has been significant negotiation at the time of grant agreement signature.

### Conducting resource tracking for GF grants

#### Categorizing costs and expenditures

From the first steps of analysis, we found that neither the routinely reported SDA nor the cost categories were useful for understanding how GF HIV/AIDS grant resources are used for investment in SRH-HIV integration. The first challenge was that there is no way to map the budgeted amounts in the Component Budget of a proposal to activities within SDAs. Proposed budgets and signed budgets can be mapped to expenditures by SDA. But as we describe, each SDA contains a multitude of activities, some of which pertain to SRH integration and some which do not. The GF also uses cost categories in proposal and signed budgets; these, however, are not prospectively used for expenditure tracking by PRs, who retro-fit expenditures to cost categories prescribed by the GF. In doing so, we found several SDAs and activities within SDAs that were categorized differently by PRs and LFAs than in the signed budgets (e.g., human resources categorized as infrastructure; health products categorized as infrastructure). Like the SDAs, the cost categories are broad, and within each is a mix of activities that either relate to SRH integration or not.

There were further problems, when we sought to track resources by cost category and SDA. When we examined the expenditure reports and costed workplans from the LFA, we found that several activities had moved from one SDA to another, or could not be identified at all in the expenditure reports. This may have been the result of grant negotiations, but there was no documentation of the specific changes. HIV and SRH stakeholders in Ethiopia all identified that the grant negotiation process had resulted in significant re-programming and refinement of activities, so that the SDAs in the signed grant agreement do not give a clear enough picture of what activities are actually being implemented. One objective of our in-depth interviews with PRs and CCM members was to try and get a clearer understanding of the differences we noted between signed agreement and expenditure reports; these showed that many activities changed from the time of the proposal until the grant agreement was signed – including an adjusted timeline – but memories were not complete, and with an absence of documentation, we did not have the scope to verify and validate what was reported from different sources.

The SDAs as articulated in grant agreements are simply too broad to allow for meaningful resource tracking of Global Fund grants, without accompanying details on activity implementation plans. Even the implementing partners in Ethiopia did not find the SDA categories to be useful – yet the grant agreement and budgets still use SDAs as the primary unit of analysis. The more accurate and useful unit of analysis for budgets and expenditures is the activity.

#### Data availability

Our findings point to the significant limitations in using the existing Global Fund grant performance monitoring systems to get a full picture of SRH-HIV integration. Although implementing partners track expenditures by activity within SDAs, and provide the LFA with activity-based expenditure reports, the GF Secretariat does not currently require PRs to report expenditures by activity for the grant performance reports (GPRs) that are part of the performance-based financing system. The GF also does not make the costed, activity-based budgets that are signed at the time of grant agreement publicly available, further hindering use of the GF Secretariat’s PBF database for useful resource tracking and expenditure analysis.

As noted in previous studies [9,10], the lack of agreed upon SRH-HIV integration–related indicators is a major hindrance to monitoring progress on integration, and this study encountered the same limitation, to the extent that it was not possible to link grant expenditures with progress on SRH or HIV-SRH integration indicators. As one example, PMTCT indicators are directly related to SRH-HIV integration and linkage, and presented the clearest opportunity for analyzing expenditures and performance achievement. However, the GPR data for the Ethiopia PMTCT grant was in numbers instead of the required percentage format, and it was unclear whether a cumulative target or period-specific target was shown. For other SRH linkage areas, specific indicators were mostly absent from the grant agreements and the GPRs. The Global Fund continues to work with partners to develop a better monitoring framework. That said, there is much room for improving definitions and formulations of the few SRH-related indicators that currently are part of the Global Fund M&E Framework.

Activity-based cost and expenditure reporting is essential for accurate resource tracking of GF grant monies. Without activity-specific grant program information, a clear picture of how Global Fund grant monies are actually being used cannot be formulated, and, as we show, SDA-based expenditure analysis cannot be interpreted with confidence. The challenges to doing activity-based analysis are not insignificant. We found that even after getting detailed data that is not publicly available, including the costed activity-based budgets from the GF Secretariat, it was necessary to collect data directly from PRs and the LFA. We also needed to interview stakeholders in Ethiopia to get a better understanding of what was actually happening with grant activities, and actually observe how SRH-HIV integration was being implemented in clinical settings, in order to more accurately categorize expenditures. Collecting data by going to country level, and then to health facility level within the country, was essential for understanding SRH-HIV integration. The lack of activity-based expenditure reporting maintained as part of the GF PBF database place severe limitations on conducting routine resource tracking for GF grants, as the resources needed to do studies using primary data collection at country level are great.

The vast amount of data available through the GF’s PBF system are tempting to use for many types of financial and performance analysis. However, users of this information need to be made aware of the serious limitations. The grant negotiation process introduces much opacity into the data, obscuring linkages between proposals, budgets, and expenditures; activity-based financial data are not made available; and, there is often a lack of relevant performance indicators that can be linked to specific activities, further limiting meaningful analysis. Other studies have been similarly frustrated by the opacity of the GF budgeting, grant negotiation, and spending records [3,5,10-12].

#### Synergistic service integration and diagonal investments of GF grants

GF grant budgets and GPRs are constructed to support monitoring of achievements in the designated grant areas: HIV/AIDS, TB, malaria, HSS, and TB-HIV. However, this is possible only using SDAs as the unit of analysis, and we show the serious limitations of this approach. In the case of trying to understand integration of synergistic services, or HSS, SDA-based analysis presents even more challenges.

Some activities were clear and easy to allocate to SRH-HIV integration and linkage areas; for example, HIV testing and counseling of pregnant women, PMTCT prophylaxis, condom distribution and other family planning services, and sexual health behavior change interventions are specific examples of critical services in both the HIV and SRH realms. PMTCT services, the primary intersection of SRH and HIV/AIDS programming for treatment and care, were also considered to be directly integration related. Health systems strengthening activities were hypothesized to support both SRH and HIV service activities, although it could not be known whether these were specifically facilitating the integration of these two programs. Interviews and discussions with key stakeholders in HIV, MNCH, SRH, and PMTCT, and observations of six health centers in Ethiopia, helped us to determine that we should consider these expenditures to be indirectly related to SRH-HIV integration. Consultations with all stakeholders confirmed that the broad SDA categories mixed activities that directly and indirectly support SRH-HIV integration; therefore, the study team worked with the LFA to obtain activity-based expenditures for more accurate categorization and estimation.

Trying to examine GF grants using information in the Secretariat’s Grant Performance Monitoring and management systems gives one a sense that there is little documentation of what is actually going on at country level. These are important barriers to formulating a better picture of how Global Fund grants support more than just HIV/AIDS, TB and malaria, through either service integration or health systems strengthening support – or to put it another way, to better understand what value is there for GF grant money. These barriers are especially important to overcome as the GF moves forward with implementing its New Funding Model [13] and the Investing for Impact strategy [2].

Diagonal financing is an important concept that supports the GF approach to investing primarily in three specific diseases [14]. This is based on the idea that programs targeting these diseases must be accompanied by activities that more broadly support the development of resilient health systems, in order to maximize long-term success, and therefore, value for money. These include investments in training and expansion of the health workforce, integration and coordination with other disease programs, strengthening supply chain, laboratories, monitoring and evaluation functions, as well as increasing access to services through health financing such as universal health coverage [14,15]. If we consider the GF’s future direction and strategic approach, the GF has recently re-affirmed its commitment to investing in HSS [16]. The GF has been investing in HSS from the beginning, both through direct grant mechanisms and diagonally through grants for the three diseases. However, it will face similar challenges to demonstrating value for HSS money as we faced with resource tracking for SRH-HIV integration. Just documenting the amount of funds investing in building resilient health systems will be difficult; looking only at HSS grants will be serious underestimation, and the SDAs of disease-specific grants mask significant amounts of HSS activities that are a challenge to assess. This will be a significant weakness as the GF seeks to demonstrate its value for money, important both for maximizing impact and for maximizing replenishments. The Global Fund should be able to estimate, on a regular basis, how its HIV/AIDs grants are supporting the relatively neglected area of reproductive health, through integration of SRH, or how HIV-AIDS grants contribute to overall health systems strengthening – among other things. The current PBF data monitoring system will not allow this without fundamental changes to reporting requirements.

## Conclusions

This study found that publicly available grant information had to be triangulated with in-country financial reports and key stakeholders regarding the grant negotiation and budget signing process, before we could reasonably categorize expenditures for analysis. Even with such detailed, triangulated information, we were unable to track resources for all the HIV/AIDS grants to SRH integration achievements, and unable to propose a replicable methodology. We believe that using SDAs as the primary organizing unit for GPR data is one of the problems, and the lack of documentation on the grant negotiation and effort to link submitted proposals with signed grant agreements is another. Limited performance indicators that are meaningful for linking expenditures with program achievements are yet another problem. A simple step to remedy the first set of financial data problems is to make existing activity-based budgets and expenditure reports publicly available.

It is not at all clear why the GF Secretariat does not require activity-based expenditure reporting, or make the costed activity budgets available in the grant portfolio database. The information exists, and is routinely verified at country level. The same situation is likely found in other countries; PRs have the motivation to monitor the activities and expenditures of their sub-recipients. But the GF Secretariat either does not maintain this information itself or wish to make it publicly available. This is a serious shortfall in transparency, one of the GF’s central guiding principles.

Despite the fact that the necessarily adaptive nature of GF grant implementation in pursuit of “country ownership” can make real-time expenditure analysis and classification challenging, this is not a reason to avoid doing it. In fact, it could be achieved in the interim by integrating costed activity workplans into the GF’s routine performance monitoring system. The new funding model and the 2012-2016 strategy present good opportunities for over-hauling the PBF system to improve transparency and to allow the GF to actually track resources expended, so they can use this information to maximize value for money.

## Appendix A

Description of Methodology for Budget Extraction, **WHO-Global Fund Sexual and Reproductive Health case study – Ethiopia***

Final detailed budgets were collected for the following grants: Round 2 RCC and Round 7, Phase 1. The “All data” tab contains all activities listed in the final, approved, detailed budgets for these grants (not publicly accessible). The tab “SDA analysis” contains activities only for those SDAs of interest in this research.

Cost categories for each activity were assigned by the PR and the global amount was recorded and reported to the Global Fund (as reflected in the Grant Agreement. PRs are not required to submit line by line analysis by cost category to the Global Fund. Therefore, we are not able to provide this data, and in our analysis, the aggregated amount by cost category may not exactly reflect the aggregated cost category amounts as listed in the grant agreement. To be able to do analysis by cost category, each activity was mapped to a cost category, as defined in the Budget Guidelines.

The “Summary” tab contains aggregates for each SDA of interest, and the share of the budget for each SDA by year. The “Pivot” tab contains totals by cost category by year, and by grant.

N.B. For RCC, the Grant Agreement amount lists the total as US $ 63 298 605, however the actual amount according to the final detailed budget is $51, 154,748. The difference (approx. US $12 mil) is due to the fact that *Activity 9.1.1. upgrading and equipping rural health facilities* was delayed from Year 1 into year 2.

*provided by the GF Secretariat.

## Additional files

Additional file 1:
**Letter of introduction used for informed consent.**
Additional file 2:
**Table a. Comparison of expenditure types for ET-708-G08-H, by SDA and by activity.** **Table b**: Expenditures by Service Delivery Area and by Activity for ETH-708-G08-H. **Table c**. Comparison of Expenditure Types for ETH-202-G03-H-00, by SDA and by Activity. **Table d**. ETH-202-G03-H-00 – Expenditures by Service Delivery Area and by Activity: January 2009 – June 2011.
