# Supplementary material for: Tracking Global Fund HIV/AIDS resources used for sexual and reproductive health service integration: case study from Ethiopia
Source: Global Health. 2015 May 27;11:21. doi: 10.1186/s12992-015-0106-z (PMC4453032; doi:10.1186/s12992-015-0106-z)
Supplement: Additional file 1: — Letter of introduction used for informed consent. [file 12992_2015_106_MOESM1_ESM.pdf]

The Global Fund
To Fight AIDS, Tuberculosis and Malaria

5 September 2011

Subject: Case study on Global Fund support to sexual and reproductive health in Ethiopia

Dear CCM members, PRs, HAPCO staff, LFA and Partners,

The Global Fund has contributed significantly to improving health outcomes for women and children through investments in HIV, TB, malaria programs and health systems strengthening. Within this context, the Global Fund Secretariat is collaborating with the World Health Organization (WHO) to undertake a case study in Ethiopia to explore the impact of Global Fund-financed sexual and reproductive (SRH) activities in the Round 2-RCC and Round 7, Phase 1 HIV grants to the HAPCO.

The case study will be based on a review of existing documentation and in-country key informant interviews with main stakeholders, including Global Fund PRs and CCM members. As a partner critical in the response to HIV/AIDS and the improvement of SRH outcomes in Ethiopia, I would be most grateful if you would kindly agree to serve as a key informant.

I take this opportunity to introduce the consultant contracted by WHO, **Dr Sangeeta Mookherji** of George Washington University in Washington D.C., USA., who will conduct the study in close collaboration with country partners. Dr Mookherjee will be in Ethiopia for in-country research from **September 13-25**, during which time she would like to meet with you to discuss the role of SRH activities and linkages to the HIV/AIDS program. The findings from the study will be shared with country partners and are intended to be used to contribute to the evidence base on integration of HIV and SRH activities and also for advocacy and communication opportunities.

We thank you in advance for your participation in the case study. We would be happy to provide any further information you may require, for which you may contact Dr Kirsi Viisainen (Kirsi.Viisainen@theglobalfund.org) at the Global Fund Secretariat.

With kind regards,

Dr Dumitru Laticevschi
Fund Portfolio Manager, Ethiopia

Copy to:

Dr Dale Huntington, Scientist, Department of Reproductive Health and Research, World Health Organisation,
Dr Kirsi Viisainen, Manager, Program Effectiveness Team, Global Fund Secretariat
Dr Sangeeta Mookherji, Assistant Professor, George Washington University
