# Supplementary material for: Tracking Global Fund HIV/AIDS resources used for sexual and reproductive health service integration: case study from Ethiopia
Source: Global Health. 2015 May 27;11:21. doi: 10.1186/s12992-015-0106-z (PMC4453032; doi:10.1186/s12992-015-0106-z)
Supplement: Additional file 2: — Table a. Comparison of expenditure types for ET-708-G08-H, by SDA and by activity. Table b: Expenditures by Service Delivery Area and by Activity for ETH-708-G08-H. Table c. Comparison of Expenditure Types for ETH-202-G03-H-00, by SDA and by Activity. Table d. ETH-202-G03-H-00 – Expenditures by Service Delivery Area and by Activity: January 2009 – June 2011. [file 12992_2015_106_MOESM2_ESM.doc]

**Supplemental data**

**Table a. Comparison of expenditure types for ET-708-G08-H**, by SDA and by activity

| Expenditure Type: | Directly Integration Related | Indirectly Integration Related | Non Integration Related | Total |
| --- | --- | --- | --- | --- |
| By SDA (LFA data*) | $4,755,920 | $6,906,651 | $0 | $11,662,571 |
|  | 41% | 59% | 0% |  |
| By SDA  (PR Data) | $6,889,224 | $16,878,968 | $0 | $23,768,192 |
|  | **21%** | 79% | 0% |  |
| By Activity  (PR Data) | $8,009,062 | $15,759,130 | $0 | $23,768,192 |
|  | **34%** | 66% | 0% |  |
| Source: LFA data, December 2011; PR data, September 2011  *LFA expenditure data differed from PR expenditure data for the period, possibly reflecting a lag in reporting from the PR to the LFA | | | | |

# Table b: Expenditures by Service Delivery Area and by Activity for ETH-708-G08-H

| **Directly Integration Related SDAs** | **Expended through June 2011**  **(% of grant total)** | **Activity** | **Directly Integration Related Y1 Expenditure** | **Indirectly Integration Related Y1 Expenditure** | **Correspondence to Y1 Agreement Budget** | **Comments**  **(Link to WHO framework)** |
| --- | --- | --- | --- | --- | --- | --- |
| **SDA 1: PMTCT** | **$6,889,224**  **(X%)** | Procure and distribute continuous supply of HIV/AIDS drugs for PMTCT prophylaxis | $1,101,840 |  | $903,382 | Related to the linkage area of integrating HIV/AIDS with maternal and infant health |
|  |  | Train staff working in Mother Neonatal &Child Health (ANC, delivery, postnatal, FP & EPI) in new PMTCT guideline | $371,108 |  | $346,697 |  |
|  |  | Provide additional training on PMTCT & delivery for HEWs | $3,394,898 |  | $3,255,039 |  |
|  |  | Conduct additional training of TBAs on PMTCT | $181,570 |  | $230,194 |  |
|  |  | Develop new & improved different IEC/BCC printed materials for PMTCT | $180,303 |  | $214,641 |  |
|  |  | Produce, TV /radio dramas on PMTCT and purchase air time for dissemination | $194,555 |  | $408,000 |  |
|  |  | Conduct pilot study of PMTCT outreach workers in pastoralist regions | $235,677 |  | $324,000 |  |
|  |  | MA1.8.1: Improve workplace programs for prevention in key public sectors | $1,229,274 |  | $1,900,000 |  |
| **PMTCT SPLIT TOTALS** | | | **$6,889,224** | **$0** |  |  |
| **PMTCT COMBINED TOTALS** | | | **$6,889,224** | | $7,581,953 |  |

| **Indirectly Integration Related SDAs** | **Expended through June 2011**  **(% of grant total)** | **Activity** | **Directly Integration Related Y1 Expenditure** | **Indirectly Integration Related Y1 Expenditure** | **Correspondence to Y1 Agreement Budget** | **Comments**  **(Link to WHO framework)** |
| --- | --- | --- | --- | --- | --- | --- |
| **SDA 2.1: Human Resources (HR)** | **$9,181,951**  **(39%)** | Recruit HIV Care AIDS (patient experts) in hospitals & health centers |  | $3,500,000 | $504,000 | Links to SRH not clear |
|  |  | Recruit data clerk for health facilities & health offices |  | $903,382 | $467,820 | Links to SRH not clear |
|  |  | Top up payment for HIV/AIDS focal person at RHB | $346,697 |  |  | Related to the linkage area of integrating HIV/AIDS with maternal and infant health |
|  |  | Accommodation services for key health professionals and night duty staff in 50 remote health centers |  | $3,255,039 | $3,000,000 | Links to SRH not clear |
|  |  | Conduct PMTCT Campaign as part of safe motherhood initiative | $230,194 |  |  | Related to the linkage area of integrating HIV/AIDS with maternal and infant health |
|  |  | Train and involve HEWS in PMTCT Service promotion and linkage of service | $214,641 |  |  | Related to the linkage area of integrating HIV/AIDS with maternal and infant health |
|  |  | Train community facilitators drawn from community (religious leaders, HIV positive, Women, Spouse) |  | $408,000 |  | Links to SRH not clear |
|  |  | Linkage between community based organizations and health facilities to increase ANC uptake | $324,000 |  |  | Related to the linkage area of integrating HIV/AIDS with maternal and infant health |
| **HR SPLIT TOTALS** | | | **$1,115,531** | **$8,066,420** |  |  |
| **HR COMBINED TOTALS** | | | **$9,181,951** | | $3,971,820 |  |

| **Indirectly Integration Related SDAs** | **Expended through June 2011**  **(% of grant total)** | **Activity** | **Directly Integration Related Y1 Expenditure** | | | **Indirectly Integration Related Y1 Expenditure** | **Correspondence to Y1 Agreement Budget** | **Comments**  **(Link to WHO framework)** |
| --- | --- | --- | --- | --- | --- | --- | --- | --- |
| **SDA 2.2: Health Management and Network Organization (HMNO)** | **$5,214,177**  **(22%)** | Construction of health centers |  | | | $3,792,274 | $0 | Related to HSS, but without a specific goal of integration |
|  |  | Train clinical mentors from hospitals, health centers & district health offices. |  | | | $201,468 | $76,636 |  |
|  |  | Establishing and equipping regional medical equipment training centers |  | | | $233,585 | $875,000 |  |
|  |  | Conduct review meeting every month |  | | | $85,365 | $67,620 |  |
|  |  | Procure SIM cards for mentors and recipients of the mentoring services. |  | | | -$5,500 | $69,496 |  |
|  |  | Conduct off site mentoring and supervision in the facilities quarterly |  | | | $47,724 | $176,400 |  |
|  |  | Operational costs for clinical mentoring & recipients |  | | | $28,093 | $50,400 |  |
|  |  | World Aids Day Celebration |  | | | $87,547 | $0 |  |
|  |  | Construction of Health Post |  | | | $743,620 | $0 |  |
| **HMNO SPLIT TOTALS** | | | **$0** | | | **$5,214,177** |  |  |
| **HMNO COMBINED TOTALS** | | | **$5,214,177** | | | | $1,315,552 |  |
| **SDA 2.3: Procurement and Supply Chain Management (PSM)** | **$2,156,408**  **(9%)** | Procure & install servers for local area network & telemedicine | **$0** | | $2,156,408 | | $4,636,000 | Related to HSS, but without a specific goal of integration |
| **PSM SPLIT TOTALS** | | | **$0** | **$2,156,408** | | |  |  |
| **PSM COMBINED TOTALS** | | | **$2,156,408** | | | | $4,636,000 |  |
| **SDA 2.4: Expand Public-Private Partnerships (PPP)** | **$4,307**  **(0%)** | Provide incentives to 10 private hospitals based on monthly report on mothers and babies who received prophylaxis for PMTCT | $4,307 |  | | | $6,160 | Related to the linkage area of integrating HIV/AIDS with maternal and infant health |
| **PPP SPLIT TOTALS** | | | **$4,307** | **$0** | | |  |  |
| **PPP COMBINED TOTALS** | | | **$4,307** | | | | $6,160 |  |

| **Indirectly Integration Related SDAs** | **Expended through June 2011**  **(% of grant total)** | **Activity** | **Directly Integration Related Y1 Expenditure** | | **Indirectly Integration Related Y1 Expenditure** | **Correspondence to Y1 Agreement Budget** | **Comments**  **(Link to WHO framework)** |
| --- | --- | --- | --- | --- | --- | --- | --- |
| **SDA 2.5: HMIS Strengthening and Monitoring & Evaluation (SDA M&E)** | **$232,792**  **(1%)** | Joint quarterly supportive monitoring from regions to implementers (Finance, GF, M&E department) quarterly including air ticket, fuel, DSA) |  | | $123,793 | $87,643 | Related to HSS, but without a specific goal of integration |
|  |  | Mapping of service providers at different levels and develop or update lists of points of distribution |  | | $27,898 | $340,149 |  |
|  |  | Strengthen the system to capture data to monitor clients satisfaction for all programs |  | | $81,102 | $113,253 |  |
| **SDA M&E SPLIT TOTALS** | | | **$0** | | **$232,792** |  |  |
| **SDA M&E COMBINED TOTALS** | | | **$232,792** | | | $541,045 |  |
| **M&E** | **$89,333**  **(0%)** | Printing of reporting formats for sectors |  | | $0 | $31,500 | Related to HSS, but without a specific goal of integration |
|  |  | Reporting of data from the selected sectors |  | | $0 | $345 |  |
|  |  | Bank Finance Charge |  | | $89,333 | $0 |  |
| **M&E SPLIT TOTALS** | | | **$0** | **$89,333** | |  |  |
| **M&E COMBINED TOTALS** | | | **$89,333** | | | $31,845 |  |

**Table c. Comparison of Expenditure Types for ETH-202-G03-H-00, by SDA and by Activity**

| Expenditure Type: | Directly Integration Related | Indirectly Integration Related | Non Integration Related | Total |
| --- | --- | --- | --- | --- |
| By SDA | $7,699,594 | $18,562,575 | $3,880,371 | $30,142,540 |
|  | 26% | 62% | 13% |  |
| By Activity | $19,776,770 | $6,485,399 | $3,880,371 | $30,142,540 |
|  | 66% | 22% | 13% |  |
| Source: LFA data, December 2011 | | | | |

**Table d. ETH-202-G03-H-00 – Expenditures by Service Delivery Area and by Activity: January 2009 – June 2011**

| **Directly Integration Related SDAs** | **Expended through June 2011**  **(% of grant total)** | **Activity** | **Directly Integration Related Y1 Expenditure** | **Indirectly Integration Related Y1 Expenditure** | **Correspondence to Y1 Agreement Budget** | **Comments**  **(Link to WHO framework)** |
| --- | --- | --- | --- | --- | --- | --- |
| **SDA 1: BCC** | **$1,970,691**  **(7%)** | MA1.1.1: Organizing continuous community conversation & dialogue on HIV/AIDS at 17,000 kebeles | $1,088,159 |  | Orig. $10,200,000  Reprog.* $1,428,000 | Assumes community conversations include integration components (e.g. promoting VCT + condom use, ANC, or FP); HEWs involved, so likely messages are integrated |
|  |  | MA1.2.1: Outreach for prevention of transmission of HIV among uniformed forces, laborers and pastoralists | $0 |  | Orig. $196,486  Reprog.* $158,326 | Assumes outreach efforts include integration messages (e.g. promoting VCT + condom use or FP); special populations are targeted |
|  |  | MA1.3.1: Support 8 local and national media to produce HIV programs and disseminate for an hour weekly for 52 weeks per year |  | $130,303 | Orig. $551,200  Reprog.* $275,600 | Need to know more about programs |
|  |  | MA1.4.1: Promote education and peer support through establishing and equipping youth centers in 100 districts over three years | $0 |  | Orig. $476,000  Reprog.* $0 | Related to “Reorientate VCT services to better meet the needs of young people, as well as of key populations” |
|  |  | MA1.5.1: Extend HIV Prevention programs from high schools to all 20,000 primary and secondary schools over the coming three yrs | $751,267 |  | Orig. $5,300,000  Reprog.* $960,000 | Related to “Reorientate VCT services to better meet the needs of young people, as well as of key populations” |
|  |  | MA1.6.1: Continue support to 11 regional AIDS information centers to produce different IEC/BCC materials for the region, youth center & school AIC and also provide information to the young & needy people in their respective regions through internet service. | $961 |  | Orig. $2,807,530  Reprog.* $2,530 | Related to “Reorientate VCT services to better meet the needs of young people, as well as of key populations” plus others depending on promotion foci. |
|  |  | MA1.7.1: Expansion of the MARCH (Modeling & Reinforcement to combat HIV/AIDS) initiative to all target populations over three years |  | $0 | Orig. $516,668  Reprog.* $0 | Links to SRH not clear |
|  |  | MA1.8.1: Improve workplace programs for prevention in key public sectors |  | $0 | Orig. $50,000  Reprog.* $50,000 | Links to SRH not clear |
| **BCC SPLIT TOTALS** | | | **$1,840,387** | **$130,303** |  |  |
| **BCC COMBINED TOTALS** | | | **$1,970,690** | | Orig.  Reprog.* |  |

| **Directly Integration Related SDAs** | **Expended through June 2011**  **(% of grant total)** | **Activity** | **Directly Integration Related Y1 Expenditure** | **Indirectly Integration Related Y1 Expenditure** | **Correspondence to Y1 Agreement Budget** | **Comments**  **(Link to WHO framework)** |
| --- | --- | --- | --- | --- | --- | --- |
| **SDA 2: Condom Distribution** | **$495,433**  **(2%)** | 2. 2 Procurement and distribution of condoms to youth centers (35,539,099 pieces) | $495,433 |  | $1,135,277 | Related to “Promote condom use for dual protection within all family planning and HIV prevention programmes” |
|  |  | 2. 3 Procurement and distribution of 9600 female condoms ( 1120 Pieces ) | $0 |  | $1,120 |  |
| **CONDOM DISTRIBUTION SPLIT TOTALS** | | | **$495,433** | **$0** | $1,136,397 |  |
| **CONDOM DISTRIBUTION COMBINED TOTALS** | | | **$495,433** | |  |  |
| **SDA 4: HIV/AIDS Counseling and Testing (HCT)** | **$4,089,481**  **(14%)** | MA4.1: Expanding HCT services to 3,303 health centers and hospitals by 2010. There will be 849 and 728 new health facilities providing HCT in 2009 & 2010 respectively as their construction will be completed in these times. | $0 |  | $0 | Related to “Learn HIV Status and Access Services” |
|  |  | MA4.2: Strengthening provider initiated counseling and testing in all 3,303 health facilities and referral linkage within the facility. | $0 |  | $0 |  |
|  |  | MA4.3: Improving HCT acceptance and up take through the active involvement of health extension workers, religious leaders and local community leaders in providing household based education on HCT, benefits of knowing one’s HIV status and being role model. | $0 |  | $0 |  |
|  |  | MA4.4: Conducting mobile HCT in pastoralists and some other areas. | $0 |  | $3,600 |  |
|  |  | MA4.5: Procurement and distribution of test kits | $4,089,481 |  |  |  |
| **HCT SPLIT TOTALS** | | | **$4,089,481** | **$0** | $3,600 |  |
| **HCT COMBINED TOTALS** | | | **$4,089,481** | |  |  |

| **Directly Integration Related SDAs** | **Expended through June 2011**  **(% of grant total)** | **Activity** | **Directly Integration Related Y1 Expenditure** | **Indirectly Integration Related Y1 Expenditure** | **Correspondence to Y1Agreement Budget** | **Comments**  **(Link to WHO framework)** |
| --- | --- | --- | --- | --- | --- | --- |
| **SDA5: ARV and Monitoring** | **$1,143,989**  **(4%)** | 6.1: Improving the capacity of hospitals on diagnosing infants and children through the provision of PCR in six selected sites | $0 |  | $20,100 | Related to “Integrate HIV/AIDS with Maternal and Infant Health” |
|  |  | 6.2: Procuring supplies and reagents for early infant diagnosis for testing 3258 in 2009, 5,825 in 2010 and 3,653 per year from 2011-2014 | $0 |  | $81,450 | Related to “Integrate HIV/AIDS with Maternal and Infant Health” |
|  |  | 6.3: Increase service delivery points by making the service available in 1353 pubic and private health facilities by 2010 | $0 |  | $0 | Related to “Integrate HIV/AIDS with Maternal and Infant Health” |
|  |  | 6.7: Procure Automated hematology, biochemistry and CD4 fax machines for 120 health facilities over three years |  | $1,143,989 | $1,680,000 | Builds ARV capacity but does not specify integration |
|  |  | 6.8.: Deploy 150 case managers over three years to provide adherence counseling, strengthening linkages/ referral, and tracing defaulters | $0 |  | 90,000 | Related to “Integrate HIV/AIDS with Maternal and Infant Health” |
|  |  | 6.9: Train 4 health care providers on ART, cotrimoxazole prophylaxis and OI management over three years |  | $0 | $0 | Builds ARV capacity but does not specify integration |
|  |  | 6.10: Ensuring continuous supply of ARV drugs for 371,192 patients in 2010, and 397,539 per year for the remaining four years (2011-2014). |  | $0 | $0 | Builds ARV capacity but does not specify integration |
|  |  | 6.11: Procure and distribute hematology, biochemistry and CD4 fax machines reagents to health facilities for ART initiation and toxicity monitoring |  | $0 | $0 | Builds ARV capacity but does not specify integration |
| **ARV SPLIT TOTALS** | | | **$0** | **$1,143,989** | $1,871,550 |  |
| **ARV COMBINED TOTALS** | | | **$1,143,989** | |  |  |
| **Total:** | **$7,699,594**  **(26%)** |  | **$6,425,301**  **(83%)** | **$1,274,292**  **($17%)** |  |  |
|  |  |  | **$7,699,593** | | **$23,109,431** |  |
| Source: LFA data, December 2011  Note: SDA expenditure totals are from LFA expenditure report for January 2009 – June 2011. Activity expenditures are from LFA Year One Expenditure Report. Although the grant has a January 1, 2009 start date, the first disbursement was made for the July – December 2010 period, indicating a year one period of July 1, 2010 – June 30, 2011. Year one expenditures match total expenditures; indicating also that only one year of spending has been completed and reported, in spite of the 2.5 year grant period.  *LFA provided reprogrammed expenditures for SDA 1, year one only, presumably reprogrammed in negotiations post grant agreement. | | | | | | |
